# Misconceptions in Physics Explainer Videos and the Illusion of Understanding: an Experimental Study

**DOI:** 10.1007/s10763-022-10265-7

**Published:** 2022-03-18

**Authors:** Christoph Kulgemeyer, Jörg Wittwer

**Affiliations:** 1 Physics Education, Department of Physics, University of Paderborn, Warburger Str. 100, 33098 Paderborn, Germany; 2 Department of Educational Science, University of Freiburg, Rempartstr. 11, 79098 Freiburg, Germany

**Keywords:** Explainer video, Misconception, Physics, Illusion of understanding, YouTube

## Abstract

Some of the online explainer videos for physics present misconceptions as scientifically correct explanations. Even so, some of these videos achieve good ratings on online platforms. A possible reason is that explainer videos with misconceptions foster an “illusion of understanding”—the mistaken belief that a topic has been understood. Misconceptions close to everyday experiences might appear more attractive than scientifically correct explanations. This experimental study (*N* = 149 physics learners with a low prior knowledge on average, sample of convenience) was conducted to explore this effect. Two explainer videos were developed to be comparable in terms of comprehensibility and duration. The explanatory approach was the only difference between the two videos. While the experimental group watched a video introducing the concept of force using misconceptions, the control group watched a scientifically correct video. Learners from the experimental group developed an equal belief of understanding, but more misconceptions and fewer scientifically correct conceptions than those from the control group. We argue that this illusion of understanding might become problematic (a) in physics instruction because students who have watched a misleading video might regard further teaching in school as irrelevant, and (b) learners might tend to rate videos including misconceptions better on an online platform like YouTube. Future studies should examine these two possible consequences.

## Introduction

Explainer videos—also referred to as, for example, explaining videos, instructional videos, or learning videos—are widely popular on online platforms (e.g., YouTube). Students watch them, for example, to prepare for exams or simply for entertainment (Wolf & Kratzer, 2015). During the COVID-19 pandemic, the importance of online explanation videos for formal education has risen (Voss & Wittwer, 2020). Such online explainer videos also have the potential to contribute to physics teaching beyond distance learning, for example, to a flipped classroom (Van Alten, Phielix, Janssen & Kester, 2019; Wolf & Kulgemeyer, 2016).

Despite their high potential, the quality of online explainer videos greatly varies (Kulgemeyer & Peters, 2016). Frequently, we come across physics videos that we think might seem problematic from a science education perspective because they present well-known misconceptions as scientifically correct explanations (Kulgemeyer, 2018) that might cause more harm than good for students’ understanding. Misconceptions might appear as “false friends” in explainer videos that create the illusion of a simple explanation that fits everyday experiences and, thus, appears seductive and convincing. Learners, however, might be unable to identify these explanatory approaches as wrong. Explainer videos based on misconceptions might, therefore, cause a so-called illusion of understanding (Chi, Leeuw, Chiu & Lavancher, 1994; Wittwer & Renkl, 2008), the phenomenon that after explanations learners sometimes tend to *think* that they have understood a topic when, *in fact*, they have not.

The present article focuses on a potential illusion of understanding after learners watched an explainer video that includes misconceptions. We developed two nearly identical videos varying just in one aspect: one is based on common misconceptions on the concept of force and the second one is scientifically correct. Both videos were developed to be comparable in terms of comprehensibility following the framework of effective science explanation videos (Kulgemeyer, 2018). In an experiment, we examined how first-year students from a teacher training college who received an explainer video with misconceptions differed from students who received a video with correct conceptions in the acquisition of an illusion of knowledge, misconceptions, and correct conceptions of force.

## Theoretical Background

### Explainer Videos

Explainer videos are short videos (usually between 5 and 10 min) aimed to explain a particular topic understandably (Findeisen, Horn & Seifried, 2019; Kulgemeyer, 2019; Wolf & Kratzer, 2015).

On online platforms, explainer videos can be found on virtually all common topics of school science curricula (Kulgemeyer & Peters, 2016). Physics videos, according to Kulgemeyer and Peters (2016), have a broad variance in explanation quality. Some of them even include misconceptions well known in science education research. For instance, the German channel “simpleclub” introduces the concept of force with examples such as “If someone is powerful, he has got a lot of force. [..] But also a car has got a lot of force.”^1^ We argue that examples like these might create the impression that force can be stored in a body, which is a well-known misconception among physics learners (Driver et al., 2014). However, as is often the case (Kulgemeyer & Peters, 2016), the comments below the video (e.g., “Thank you. That is much better than what my teacher does.”) and the video’s popularity (nearly 600,000 views as of June 2021, 7441 likes compared to 153 dislikes) do not reflect that the video might be problematic. There are probably numerous other examples of such physics explainer videos on YouTube (Kulgemeyer & Peters, 2016) because anyone can upload content and there is no quality control.

Numerous studies demonstrate the general potential of explainer videos for learning. For example, Lloyd and Robertson (2012) showed that explainer videos enhance the learning of statistics compared to print media. In a review of recent research, Kulgemeyer (2018) developed and empirically tested a framework to improve the effectiveness of science explainer videos based on evidence from studies on instructional explanations (Geelan, 2012; Kulgemeyer, 2019; Wittwer & Renkl, 2008). The framework is in line with other frameworks on the quality of explainer videos like Brame (2016) and Findeisen et al. (2019). It consists of seven factors that have been empirically shown to influence the effectiveness of explainer videos: (1) the structure of the video (Seidel et al., 2013), (2) the adaptation to a group of addressees (Wittwer & Renkl, 2008), (3) the use of appropriate tools to achieve adaptation (Kulgemeyer & Schecker, 2009, 2012, 2013), (4) minimal explanations that keep the cognitive load low (e.g., Anderson et al., 1995), (5) highlighting that the explanation itself is relevant to the learner and what the most relevant parts are (Acuña et al., 2011), (6) providing learning tasks (Webb et al., 2006), and (7) a focus on a scientific principle (Renkl, Wittwer, Große, Hauser, Hilbert, Nückles & Schworm, 2006). These seven factors can be influenced by using different features (Table 1). Overall, 14 features are highlighted as particularly important to impact the effectiveness of explainer videos. The present study utilized this framework to develop two videos that possess similar comprehensibility.

**Table 1:** Framework for effective explanation videos (from Kulgemeyer (2018))

*Factors*	*Feature*	*Description*
Structure	1. Rule-example, example-rule	Rule-example structure if the learning goal is factual knowledge. Example-rule structure if the learning goal is a routine or procedural knowledge
	2. Summarizing	The video summarizes the explanation
Adaptation	3. Adaptation to prior knowledge, misconceptions, and interest	The video is adapted to a group of addresses and their potential knowledge, misconceptions, or interests. To do so, it uses the “tools for adaptation.”
Tools for adaptation	4. Examples	The video uses examples to illustrate a principle
	5. Analogies and models	The video uses analogies and models that connect the new information with a familiar area
	6. Representation forms, demonstrations	The video uses representation forms and/or demonstrations
	7. Level of language	The video uses a familiar level of language
	8. Level of mathematization	The video uses a familiar level of mathematization
Minimal explanation	9. Avoiding digressions	The video focuses on the core idea, avoids digressions and keeps the cognitive load low
	10. High coherence	The video connects sentences with connectors, especially “because.”
Highlighting relevancy	11. Highlighting relevancy	The video explicitly highlights why the explained topic is relevant to the explainee
	12. Direct addressing	The explainee is addressed directly, e.g., by using the second-person singular
Learning tasks	13. Learning tasks	The video describes learning tasks the explainees can engage in actively
Principles	14. New, complex principle	The video focuses on a new science principle that is too complex to understand by self-explanation

### The “Illusion of Understanding”

An “illusion of understanding” occurs when learners assume to have understood a topic although they actually have not (Wittwer & Renkl, 2008). In this case, learners overestimate the knowledge they have acquired from learning. Research shows that this phenomenon is rather the rule than the exception (e.g., Dunning et al., 2003; Prinz, Golke & Wittwer, 2018). Regarding the chosen explanatory approach, we are not aware of research comparing the understanding of scientifically correct videos and videos with misconceptions. However, in the context of online explainer videos, previous research on general videos suggests four factors that might contribute to an illusion of understanding after watching an explainer video:

First, when confronted with things being explained such as in an online explainer video, learners seem to be particularly prone to overestimate how much they understand. Rozenblit and Keil (2002) call this phenomenon the “illusion of explanatory depth.” Such a phenomenon is likely to occur in understanding natural phenomena such as in physics because people erroneously assume that understanding a phenomenon at one level (e.g., level of observation) automatically leads to understanding this phenomenon at a deeper level (e.g., level of underlying mechanisms).

Second, when watching an online explainer video, learners process visual information in the form of motion pictures. Research demonstrates that such pictures can influence feelings of how much is understood (Salomon, 1984). For example, Lowe (2003) showed that graphical animations can contribute to an illusion of understanding when they attract a learner’s attention to information that is not relevant for learning. Jaeger and Wiley (2014) suggest that the effect of not being able to distinguish “between their feelings of efficacy and their actual level of understanding and effort, […] could be leading them [the learners] to make inaccurate judgments about their comprehension when images are present.” (p. 69). Wiley (2019) calls this a “seduction effect” of pictures. In addition, Paik and Schraw (2013) found an effect of learning with animations on the development of an illusion of understanding.

Third, watching is often the only activity that learners engage in when learning with explainer videos. Without undertaking further learning activities, however, there is no opportunity for learners to become aware of their illusion of understanding. According to Mayer et al. (2020), generative activities are an important way to increase the effectiveness of learning from videos. Such generative activities provide learners with cues that they can use to form a more realistic picture of their understanding (e.g., Prinz, Golke & Wittwer, 2019). Research shows that, for example, asking learners to explain newly learned content (e.g., Fernbach et al., 2013) can be useful to confront learners with their limited understanding.

Fourth, in case learners acquire faulty knowledge when learning from an online explainer video, remedial explanations that explicitly highlight a misunderstanding might be helpful (Roelle, Berthold & Renkl, 2014; Sánchez, García-Rodico & Acuña, 2009). However, when learners are not aware of which aspects of the remedial explanations address their faulty knowledge, they might perceive such explanations as being irrelevant. In this case, their limited understanding remains unchanged (Acuña et al., 2011).

Prinz et al. (2018) argues that videos that (unintentionally) contain misconceptions can make learners particularly susceptible to unwarranted feelings of understanding. In addition, Yates and Marek (2014) found that students usually finish an introductory biology course being more confident about their content knowledge while also holding more misconceptions; teachers and their own misconceptions were identified as potentially being the source of these misconceptions. This is partly because misconceptions are often based on personal experience and, thus, appear as attractive alternative explanations (cf. Kuczmann, 2017). In this case, we would expect that learners accept misconceptions as explanations of scientific concepts because they might give them the illusion of understanding, resulting in the beliefs that the video contained (a) a scientifically correct explanation, (b) a high-quality explanation in terms of comprehensibility, and the beliefs that the learners themselves (c) understood the concept, and (d) do not require further instruction on this topic. Such explainer videos might even result in a more firmly established misconception (cf. Yates & Marek, 2014). In the present article, we use these four criteria to characterize an illusion of understanding that is in line with our definition of an illusion of understanding. Additionally, to form an *illusion* of understanding (and not just a correct belief of understanding), individuals must hold these four beliefs—despite all four being untrue.

The illusion of understanding hypothesis is similar but at the same time broader than just comparing the feeling of learning with actual learning (e.g., Deslauriers et al., 2019). For instance, Paik and Schraw (2013) understand the illusion of understanding as to the effect that people (1) perceive a multimedia presentation to be easier and (2) overestimate their understanding. Thus, their concept of an illusion of understanding includes aspects of the (1) material (1) and the (2) learning individual. Our four criteria are in line with this previous research on the illusion of understanding. Just as in the study of Paik and Schraw (2013), our criteria (a, scientifically correct explanation) and (b, high perceived comprehensibility of the explanation) also describe aspects of the material (in our case explainer videos), while our aspects (c, feeling of understanding) and (d, no further instruction required) cover aspects of the learning individual.

### Preconceptions and Misconceptions on Force

Briefly, a misconception is a “belief that conflicts with currently accepted scientific explanations” (Tippett, 2010, p. 953). The importance of learners’ ideas about physical concepts or their alternative frameworks is one of the most prominent research topics in science education overall (Duit, 2009). Conceptual change remains one of the most prominent frameworks to understand the learning of science (Duit & Treagust, 2012). It was introduced by Posner et al. (1982) and has undergone several changes in the following years (Duit & Treagust, 2012). Key ideas are that students already hold conceptions about the physical world before attending the class, their ideas often differ from a scientific view, and they are hard to change (Duit & Treagust, 2012). Moreover, science teaching itself sometimes contributes to developing ideas that deviate from a scientific view (Duit & Treagust, 2012). These ideas have been called—among other things—misconceptions or preconceptions. The terms preconception and misconception are often treated as synonyms (as in the present article), although sometimes preconceptions are understood as ideas about the physical world that learners possess *before* formal science education while misconceptions are ideas that *result* from formal science education (Vosniadou, 2012).

For many concepts in physics, misconceptions stem from everyday language and everyday experiences. For example, following Newton’s second law, “force” is understood as *F* = *ma*; the force *F* on an object results in its acceleration *a*. However, a common misconception on this key concept of classical mechanics is that it takes a force to keep an object at a constant velocity, and if there is no external force acting on that object, it might “store force” to keep moving until this “force” is depleted (Clement, 1982; Schecker & Wilhelm, 2018, p. 73). This misconception accords well with observations of everyday phenomena: a bicycle stops moving once the cyclist stops pedaling. From a physics point of view, the “driving force” on the bicycle (resulting from the interaction of the ground and the bike following Newton’s third law) is not the only force on the object. The force of friction on the bike needs to be canceled out by the driving force to keep the bike moving with a constant velocity—in that case, the resulting force on the bicycle, therefore, is zero.

The aforementioned misconception that force can be stored in an object (Driver et al., 2014; Schecker & Wilhelm, 2018, p. 73) is a prominent one and also referred to as an “impetus view” on force (e.g., Eckstein & Shemesh, 1993). This misconception is particularly predominant among German-speaking physics learners, which the present study focuses on. The German word for “force” (Kraft) in everyday language is used nearly interchangeably with the words for “power” and “energy” (Schecker & Wilhelm, 2018). From a physics point of view, a force acts on an object which results in acceleration. However, students often have the misconception that every moving object “has force,” that this force is correlated with its mass and velocity, and that this force is depleted once the object stops being in motion. Of course, there are many more misconceptions on the concept of force documented in the literature (cf. Alonzo & Steedle, 2009). However, the two misconceptions, “force is always needed to keep a body moving” and “force is stored in an object,” are very close to everyday observations and very common (Schecker & Wilhelm, 2018). Therefore, we will use these two misconceptions in this study as a basis for the content covered by the videos.

### Research Question

Misconceptions might foster an illusion of understanding because they are, among other things, often based on personal experience and, thus, might appear as attractive alternative explanations. As already described, Yates and Marek (2014) found that students often leave their introductory biology courses with even more misconceptions than before, while being convinced that their content knowledge is sufficient; their teachers’ own misconceptions might play a role in that. We wanted to research whether misconceptions in explainer videos foster an illusion of understanding. In addition, the results of Yates and Marek (2014) might suggest that students who watch an explainer video containing misconceptions might develop more misconceptions and less scientifically correct content knowledge compared to students who watch a scientifically correct explainer video, which would make sense because it matches the learning opportunities. Based on the reported studies in the theoretical background, our research question was as follows:

- Research question 1: *Does an explainer video with misconceptions lead to a greater illusion of understanding, more misconceptions, and less conceptual knowledge than an explainer video with scientifically correct conceptions?*

For the present study, we conceived of an illusion of understanding when learners have the mistaken impression that an explanation is (a) scientifically correct and (b) comprehensible. In addition, they should draw the mistaken conclusions that (c) they have understood the explained concept, and (d) they do not require further instruction on the topic. In a nutshell, we tested for the mistaken belief that an explanation was good and resulted in understanding. This is close to the concept of an illusion of understanding used by Paik and Schraw (2013).

We wanted to ascertain whether including misconceptions in the video fosters the illusion of understanding. Therefore, we compared the experimental group (“misconception group”) and control group (“conceptual knowledge group”) for differences in the four criteria. Of course, only the experimental group learned the misconception, while the control group learned scientifically correct knowledge. Therefore, the latter might rightfully assume that the four criteria are true. Thus, we compared the groups for their belief of understanding; however, for the experimental group, this belief of understanding would be an illusion. We assumed that the presence of misconceptions leads to a higher belief of understanding than scientifically correct knowledge because they are often based on personal experience and might appear more convincing (see above). This leads to the first hypothesis:

- *Hypothesis 1: *The belief of understanding is higher after watching an explainer video that includes misconceptions than after watching a scientifically accurate explainer video.

The second and third hypotheses focused on the comparison between the scientifically accurate video and the video including the misconceptions regarding learning gains. We expected that watching the video containing the misconceptions would lead to more misconceptions and less conceptual knowledge than watching the scientifically correct video. As mentioned above, that would be in line with the results of Yates and Marek (2014) and also plausible because it matches the learning opportunities.

- Hypothesis 2: Beginner physics learners acquire more misconceptual knowledge from an introductory explainer video containing misconceptions than from a scientifically correct video.
- *Hypothesis 3: *Beginner physics learners acquire less conceptual knowledge from an introductory explainer video containing misconceptions than from a scientifically correct video.

## Method

### Procedure

**Fig. 1:**
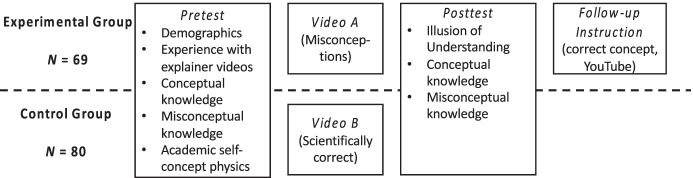
Experimental design and procedure of the study

The study followed an experimental design (Fig. 1). Two explainer videos were developed—one presenting common misconceptions as an alternative explanation and a scientifically correct one. Both videos met standards of explanatory quality by applying the framework of Kulgemeyer (2018), a rating of the explanatory quality, and criteria of text comprehensibility (see the “Design of the Explainer Videos” section).

The physics learners used their own laptops. We randomly assigned the learners to one of the groups using a random generator. Afterwards, we administered a link to an online testing environment we programmed on a WordPress base for this purpose. Learners took the tests individually online, sitting separately at their homes, while being supervised using an online meeting conference tool that we also used for physics lectures and online exams. They were not allowed to access additional information or browse the Internet. The testing time was limited to 60 min; the solutions for the knowledge tests could not simply be found online because the test items matched the content of the developed explainer videos. This makes it unlikely that learners had access to unauthorized aid.

Their experience with online explainer videos and their academic self-concept in physics were measured. Their prior knowledge on the concept of force was tested in two ways: we tested for the “conceptual knowledge” and the “misconceptual knowledge” (cf. the “Instruments” section). The first evaluates how well the learners handle the scientifically correct knowledge, and the second how “well” they solve items based on the misconception (or how much they are convinced by the misconception).

Following the pretest, the experimental group watched the video with the misconceptions and the control group watched the video with the scientifically correct explanation.

The posttest measured the four criteria for an illusion of understanding (see the “Research Questions” section). Additionally, it included scales for the conceptual and the misconceptual knowledge (the same test as in the pretest).

To fulfill ethical standards, the experimental group received further instruction after the experimental study. We explained that the video they watched contained incorrect information while presenting an attractive misconception as the correct explanation. Not only did this group receive the correct explanation afterwards, but they also participated in a group discussion on the topic that these kinds of explanations can be found on YouTube in explainer videos with millions of views. The key message was as follows: be critical with explainer videos from unknown sources.

### Participants

The sample comprised of German-speaking students from one single introductory physics course (a lecture with about 150 students each year) at one single German university that is mandatory for first-semester pre-service elementary teachers. Explainer videos have their best place at the beginning of teaching sequence when principles get introduced (Kulgemeyer, 2018) and that is the case with the learners in the sample. Usually, these learners have very little knowledge of physics as it is an optional subject in the last 3 years of school. They usually have limited physics experience in school. Therefore, they were chosen as a convenience sample for beginning physics learners before getting introduced to the concept of force.

The entire group (*N* = 185) was invited to participate. Participation was voluntary. A random generator was used to assign learners to the groups. Thirty-six learners decided not to participate because they did not allow their data to be collected. Due to German law, their data have been erased and, thus, cannot be accessed to compare their characteristics to those who participated. This probably leads to unequal numbers of learners assigned to the two experimental conditions. In total, 149 learners (126 female, 23 male, 0 diverse; *n*_*E*_ = 69 in the experimental group, and *n*_*C*_ = 80 in the control group) participated in the study. As expected, their experience was low; 115 of the learners had not enrolled in a physics course in the last 3 years of school (the academic preparation phase that leads to the German “Abitur” [equivalent to A levels]), 26 of them had taken a basic physics course, and 8 took physics as a major course for the German Abitur.

The participants differed in their age and education from the population of all physics learners before their introduction to the concept of force. Therefore, the results cannot be generalized to, for example, learners at schools. However, since the participants had limited physics experience at school and had just started their academic teacher training (thus, having just left school), the results of our study should at least provide first insights into this target population. Following Gollwitzer, Eid and Schmitt (2017, p. 92), using university students as a sample is appropriate when the characteristics in which they differ from the entire possible population are not likely to greatly influence the dependent variables. This should be the case with the differences in age and gender (the participants are mostly female). Prerequisites for these first insights are low prior knowledge (as the target group are learners before being formally introduced to the concept) and limited self-concept. Self-concept is associated with learning gains, and many beginner learners have a low self-concept (e.g., Marsh, & Yeung, 1998). In addition, studies have shown that learners often have high experience with explainer videos (Wolf & Kratzer, 2015). This should be the case for the participants as well, and was included as a control variable. If the tested hypotheses of the present study were true, they should be confirmed with our sample, even though we are aware that confirming the hypotheses with our sample of convenience should not be confused with a generalization of the results.

### Design of the Explainer Videos

The video offering an explanation based on the misconceptions explained the concept of force relying on the two misconceptions “force is always needed to keep a body moving” and “force is stored in an object.” The other video explained the concept of force correctly as the reason for an acceleration (*F* = *ma*) and that it does not require a force to keep an object moving (Newton’s first law).

The explanatory approach (misconception versus scientifically correct) was the only difference between the two videos. Both videos had a similar duration (video with misconceptions 2:12 min, video with scientifically correct explanation 2:23 min), and the design was identical (the same visuals and animations using PowerPoint and stick figures). The voiceover was performed by the same male person. To ensure that both videos were comparable in terms of language complexity, we calculated the readability scores of the texts. We used the readability coefficient LIX for this purpose because it works well with the German language (Kulgemeyer, 2018). Both scripts were found to be comparable with scores indicating a low complexity (*LIX*_scientifically correct_ = 35.60; LIX_misconceptions_ = 33.20), even though the language of the scientifically correct video might have been more formal. The videos were presented as part of the online survey tool; therefore, additional information, such as likes/dislikes, was not accessible to the learners.

Both videos were of comparable quality to allow the comparison of the explanatory approaches. Based on the criteria of Kulgemeyer (2018), both videos followed a rule-example structure and included a summary in the end. Both videos used the same example and representation forms, level of mathematization, and level of language. Both videos focused on the concept (minimal explanation), avoided digressions, and had high coherence by connecting the sentences with connectors. Both videos used prompts to highlight relevant parts and addressed the explainee directly. Both videos ended with the same learning task, even though the learners had just enough time to think about the learning task (about 5 min for each group). Finally, both videos explained the same complex principle, however, using different approaches.

Table 2 provides a brief overview of the scripts of the videos. They have been translated because the explainer videos were produced in the German language.

**Table 2:** Comparison of the explainer videos (parts of the scripts, translated from German language). The used representation forms are not included in Table 2, and the used examples are just summarized briefly

*Video with correct scientific explanation*	*Video including misconceptions*
*(Pointing out that force is an important concept in physics, giving overview of the video.)* *In a nutshell, force is the reason why an object accelerates. A force always acts with a certain “strength” in a particular direction. This means that an object will not accelerate if no force acts on it* *Of course, in that case, the object may rest, but it may also move with a constant velocity. In both cases, the object would not accelerate and, therefore, in both cases no force acts on the object* *How strong the acceleration is, given that a certain force is acting on an object depends on the mass of the object. If it has a lower mass, the acceleration will be higher. If it has a higher mass, the acceleration will be lower* *This idea on force can be used to describe all kinds of moving objects* *(Example: Applying this idea on the example of a golf ball that stops moving after it gets hit because of the force of friction.)* *Let us sum up:* *•A force causes an acceleration of an object in a certain direction* *•It does not matter if the object rests or moves—if not force acts on it, it does not accelerate* *(Ending the video with a learning task: use the explained ideas to explain why a bicycle accelerates if you go downhill.)*	*(Identical introduction: see left column)* *In a nutshell, force is a physics concept used to explain why an object moves. Of course, it depends on how heavy the object is and how fast it moves. Generally speaking: an object always has a high force if it is fast and heavy* *If you compare two objects with the same velocity, the one with the higher mass also has the higher force* *If an object has no more force, it cannot move and rests. If you see an object resting, you know that it has no force stored* *This idea on force can be used to describe all kinds of moving objects* *([Example: Applying this idea on the example of a golf ball that stops moving after it gets hit once the stored force is depleted.)* *Let us sum up:* *•A body always has a high force if it is fast and heavy* *•Without force, an object will not move at all* *(Identical learning task (see left column))*

### Instruments

#### Control Variables

To control for background variables, we administered a questionnaire consisting of questions about gender, final school grade (“Abiturnote”), experience with explainer videos (5-point Likert scale ranging from 1 (totally agree) to 5 (totally disagree), e.g., “I have often watched explainer videos on topics from school or university”), and academic self-concept in physics (5-point Likert scale, six items, α = 0.90, e.g., “I get good grades in physics”).

#### Belief/Illusion of Understanding

To characterize the learners’ illusion of understanding, we applied four criteria. Individuals should have the mistaken impression that the explanation was (a) scientifically correct, and (b) comprehensible. Also, they should draw the mistaken conclusions that (c) they have understood a concept, and (d) they do not require further instruction on this topic. The criteria (a) and (b) concern the explanation itself, and (c) and (d) concern the conclusions the individuals draw from the explanation about their understanding.

For a content valid assessment, all four criteria need to be contained in the scale for the illusion of understanding. We addressed the criteria by asking for agreement with statements on a 5-point Likert scale (1 (totally agree), 5 (totally disagree); sample items, criterion (a), the video was scientifically correct; (b) The examples used in the video were well-chosen; (c) I understand what force means in physics; (d) I do not need to learn more on the concept of force to fully understand it). For criterion (b), the aspects that influence the comprehensibility of an explainer video are based on the framework of Kulgemeyer (2018) (e.g., “the examples used in the video were well-chosen”).

The final scale consisting of items for all four criteria was found to be reliable (α = 0.75, 10 items). We want to highlight that these four criteria just constitute an *illusion* of understanding in case all of them are untrue. Otherwise, the learners might just hold a correct belief of understanding.

#### Conceptual and Misconceptual Knowledge

We tested for conceptual knowledge (scientifically correct knowledge) and misconceptual knowledge (knowledge that would be correct if the misconception was correct—or, in other words, the degree of how much a student holds a misconception). The test consisted of nine items, each of which including a situation or a statement that can be explained by either a misconception or a scientifically correct concept.

**Fig. 2:**
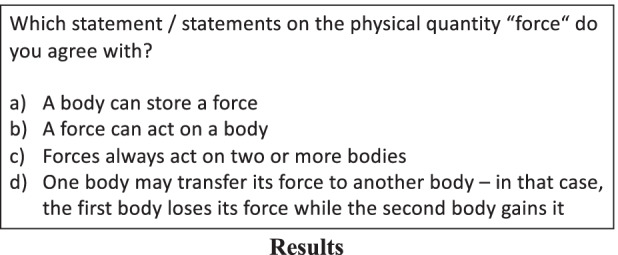
Sample item for conceptual knowledge and misconceptual knowledge (translated from the German language)

All items were multiple-choice with two of the options being the scientifically correct solution and two of the options being a solution one would pick when holding a misconception on force. All the questions could be answered independently of one another. The four answers to each question are independent aspects of the concept of force. Many test inventories (such as the Force Concept Inventory [Hestenes et al., 1992] which we used as a role-model) work similarly using options that would be used when holding a misconception as distractors for multiple-choice items. However, while the misconceptual knowledge would usually simply be counted as incorrect in studies on physics misconceptions, it is a construct with actual learning opportunities for the experimental group in our study. Figure 2 shows a sample item. In the case of the item in Fig. 2, the correct solution based on the scientifically correct concept would be (b) and (c), while someone holding the misconception that force can be stored should answer (a) and (d). The scales were calculated by summing up all scientifically correct answers (scale for conceptual knowledge) and all answers indicating a misconception (scale for misconceptual knowledge). Overall, both scales were found to be reliable (conceptual knowledge, α = 0.71; misconceptual knowledge, α = 0.85). Since it is likely that watching explainer videos without further instruction only helps to develop declarative knowledge on force (as shown by Kulgemeyer, 2018), it was necessary to keep the items as close as possible to the learning opportunities of both videos. We, therefore, ensured that learning opportunities for all items occurred in both videos by comparing the items and the scripts. Furthermore, since the explainer videos are a first introduction to the concept of force and the test items mirror the learning opportunities in these videos, the test instrument was designed for learners with low prior knowledge.

## Results

None of the variables examined in this study violated the assumptions of a normal distribution and equality of variances. To analyze the data, we used ANCOVAs. Table 3 shows the descriptive statistics of the study variables.

**Table 3:** Descriptive statistics of study variables

	Experimental group	Control group	
	*M (SD)*	*M (SD)*	Range
*Knowledge*			
Conceptual knowledge (pre)	0.48 (0.20)	0.45 (0.19)	0–1
Misconceptual knowledge (pre)	0.34 (0.16)	0.33 (0.16)	0–1
Conceptual knowledge (post)	0.50 (0.19)	0.63 (0.18)	0–1
Misconceptual knowledge (post)	0.65 (0.17)	0.35 (0.17)	0–1
*Illusion of understanding*			
Belief/illusion of understanding scale	1.66 (0.45)	1.71 (0.42)	1–5
*Controlvariables*			
Final school grade	2.14 (0.69)	2.34 (0.59)	1–6
Academic self-concept physics	3.19 (0.74)	3.31 (0.76)	1–5
Experience with explainer videos	1.96 (1.06)	1.83 (0.99)	1–5

*M* means, *SD* standard deviation

### Control Variables

Regarding the pretest scores, there was no significant difference between the control group and the experimental group for either their misconceptual knowledge (*F*(1,147) = 0.40, *p* = 0.53) or their scientifically correct conceptual knowledge (*F*(1,147) = 1.06, *p* = 0.31). The groups did not differ in gender (χ^2^(1) = 1.03, *p* = 0.31), their final school grade (*t*(92) = 1.48, *p* = 0.14), their academic self-concept in physics (*t*(147) = 1.00, *p* = 0.32), and their experience with explainer videos (*t*(147) =  − 0.78, *p* = 0.44). The control variables indicate the equivalence of the two groups. Additionally, they meet the criteria described in the “Participants” section for first insights into the population of beginner physics learners before being introduced to the concept of force.

The participants did not differ from the sample of students that started their studies one year later (*n* = 217) in their gender (χ^2^(1) = 1.76, *p* = 0.18), their final school grade (*t*(244) =  − 1.58, *p* = 0.12), or their academic self-concept in physics (*t*(360) = 1.32, *p* = 0.19). This suggests comparability of the participants with beginner physics learners in general in an academic primary teacher education environment, at least at this university.

### Descriptive Statistics

Table 3 presents descriptive statistics of the study variables for both the experimental and the control group.

Research question 1: Does an explainer video with misconceptions lead to a greater illusion of understanding, more misconceptions, and less conceptual knowledge than an explainer video with scientifically correct conceptions?

- Hypothesis 1: Illusion of understanding higher after video with misconceptions

**Fig. 3:**
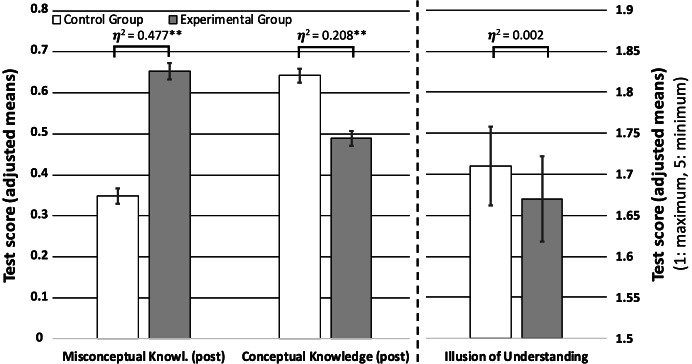
Comparison of the experimental group and the control group regarding their misconceptual knowledge, scientifically correct knowledge, and their illusion of understanding. Given are adjusted means from the ANCOVAs (for the knowledge tests, from 0 to 1; for the illusion of understanding, ranging from 1 to 5 where 1 indicates a maximum belief of understanding and 5 no belief of understanding) and the standard error of the mean. The partial η^2^ from the ANCOVAs is also given

Table 3 shows that the experimental group and the control group differ slightly in their pre-test results. To test the effect on the scale of “belief/illusion of understanding,” we used analysis of covariance (ANCOVA) adjusting for the pre-test results (conceptual knowledge and misconceptual knowledge). Regarding the scale for the belief/illusion of understanding (*F*(1,145) = 0.318, *p* = 0.57, partial *η*^2^ = 0.002), there was no statistically significant difference between both groups. Both groups scored lower than the neutral score of 3 and, thus, have a high belief of understanding (Fig. 3). This result is not fully in line with hypothesis 1.

- Hypothesis 2: More misconceptual knowledge after video with misconceptions

To test the effect on the scale of misconceptual knowledge, we used ANCOVA adjusting for the pre-test results on misconceptual knowledge. We found a statistically significant difference between the groups (Fig. 3). The experimental group achieved significantly more misconceptual knowledge with a large effect (*F*(1,146) = 133.00, *p* < 0.001, partial *η*^2^ = 0.477). This result is in line with hypothesis 2.

- Hypothesis 3: Less conceptual knowledge after video with misconceptions

To test the effect on the scale of misconceptual knowledge, we used ANCOVA adjusting for the pre-test results on conceptual knowledge (Fig. 3). The control group significantly outperformed the experimental group with a large effect (*F*(1,146) = 38.43, *p* < 0.001, partial *η*^2^ = 0.208). This result is in line with hypothesis 3.

## Discussion

The goal of the study was to gain a better understanding of potentially harmful effects of explainer videos based on misconceptions on students’ learning. To ensure internal validity, the learners were randomly assigned to two experimental conditions. Two explainer videos were developed to be comparable in terms of comprehensibility and duration. The explanatory approach was the only difference between the two videos. They had to work individually. The experimental group watched a video introducing the concept of force using misconceptions, and the control group watched a scientifically correct video. The effects on the acquisition of conceptual knowledge, misconceptual knowledge, and the development of an illusion of understanding were measured.

After watching the videos, the experimental group had an equally developed belief of understanding (hypothesis 1), outperformed the control group in misconceptual knowledge (hypothesis 2), and had less developed conceptual knowledge (hypothesis 3).

After watching the video including misconceptions, learners scored high on the belief of understanding scale. This mirrors the findings of Yates and Marek (2014), who found that students can be very confident about their knowledge while still holding many misconceptions. However, it makes no difference whether the learners watched the scientifically correct video or the video including the misconceptions—both groups have an equally high belief of understanding. Thus, the findings are not fully in line with hypothesis 1. However, since the experimental group learned misconceptual knowledge and had significantly less developed scientifically correct conceptual knowledge, just for this group, this is an illusion of understanding. The results point to the fact that learners who watch an explainer video, including misconceptions close to everyday experiences, have an illusion of understanding that matches the belief of understanding a learner has after watching a scientifically correct video, even though they have lower conceptual knowledge.

The results are in line with hypotheses 2 and 3. Both groups achieved significant learning gains matching their respective learning opportunities. We regard this match between learning gains and learning opportunities as an argument for the validity of the results because the test instruments were developed to reflect the learning opportunities (cf. Yates & Marek, 2014).

### Study Limitations

Our study has some limitations. The sample consisted of pre-service elementary teachers at the beginning of their bachelor’s program with, on average, a low level of physics knowledge (based on the pre-test results), and limited physics experience in school. They started learning the concept of force with the videos in the present study. The sample is not representative of all physics learners starting to learn the concept of force; for example, their age and education differ from learners at school, who are introduced to the concept of force. A convenience sample was chosen because it was not possible to randomly select students from the population of all beginner-physics learners starting to learn the concept of force. In addition, matching the members of the sample to a set of important population characteristics was not possible because these characteristics are unknown. The data suggest that they are representative of pre-service elementary teachers at the beginning of their bachelor’s program at this university in general. However, the sample meets important criteria that allow us first insights into the target population, including low academic self-concept in physics, limited physics experience in school, and, on average, low prior knowledge (no ceiling effect in the conceptual knowledge instrument that has been designed for low content knowledge). The variables in which the sample differs from learners at school (e.g., age) are not expected to have a high impact on the dependent variables of this study, but the choice of the sample is still a limitation for the generalization of the results.

Also, the topic was limited to misconceptions on force and misconceptions close to everyday experiences. For misconceptions on other physics concepts that do not play such a prominent role in everyday life, the results might be different. Also, it is unclear whether or not the perception of explainer videos as being scientifically correct has been influenced by the study context. The learners were informed that the material is authentic for online explainer videos, but maybe their perception that the videos are scientifically correct is influenced by the use of the videos in a study.

### Study Implications

Despite these limitations, the study suggests several implications. We regard the illusion of understanding in the experimental group as a potential problem. The perceived relevance of an explanation affects its effectiveness (Acuña et al., 2011; Kulgemeyer, 2019). If learners watched a video including misconceptions on the force concept and afterwards believed that they understood the topic and do not require further instruction, they might perceive further explanatory attempts by their teachers as redundant and irrelevant. This would mean that it might be hard for these teachers to cognitively activate their students—a major prerequisite for successful instruction (Dorfner et al., 2017). This should be examined more in detail in future studies. Also, our results would be in line with prior research indicating that video and animation have “seductive effects” that foster an illusion of understanding (e.g., Jaeger & Wiley, 2014; Lowe, 2003; Paik & Schraw, 2013; Salomon, 1984). The control group might overestimate their knowledge as well (just not as much as the experimental group). Maybe the seductive effects of video, in general, are larger than the effects of the presence of misconceptions, however, that can only be examined in future studies.

Based on the results, we developed the hypothesis that learners might rate the video including misconceptions good on an online platform—and maybe even better than the scientifically correct one. This might result in enhancing the visibility of videos with misconceptions as they may get more prominently featured on searching for videos about a particular concept. Kulgemeyer and Peters (2016) already demonstrated that neither likes nor views reflect explaining quality, and the described hypothesis might contribute to explain this. However, future studies simulating the rating process on an online platform are required to investigate this hypothesis.

## Data Availability

Data available on request.

## Footnotes

1 https://www.youtube.com/watch?v=xwsAyQGo9dQ (from 0:43 min on, accessed on 18 June 2021, translation by the authors).
